# Urinary proteomic signatures associated with β-blockade and heart rate in heart transplant recipients

**DOI:** 10.1371/journal.pone.0204439

**Published:** 2018-09-24

**Authors:** Qi-Fang Huang, Jan Van Keer, Zhen-Yu Zhang, Sander Trenson, Esther Nkuipou-Kenfack, Lucas N. L. Van Aelst, Wen-Yi Yang, Lutgarde Thijs, Fang-Fei Wei, Agnieszka Ciarka, Johan Vanhaecke, Stefan Janssens, Johan Van Cleemput, Harald Mischak, Jan A. Staessen

**Affiliations:** 1 Studies Coordinating Centre, Research Unit Hypertension and Cardiovascular Epidemiology, KU Leuven Department of Cardiovascular Sciences, University of Leuven, Leuven, Belgium; 2 Center for Epidemiological Studies and Clinical Trials and Center for Vascular Evaluations, Shanghai Institute of Hypertension, Shanghai Key Laboratory of Hypertension, Ruijin Hospital, Shanghai Jiaotong University School of Medicine, Shanghai, China; 3 Division of Cardiology, University Hospitals Leuven, Leuven, Belgium; 4 Department of Cardiology, Shanghai General Hospital, Shanghai, China; 5 Mosaiques Diagnostics GmbH. Hannover, Germany; 6 BHF Institute of Cardiovascular and Medical Sciences, University of Glasgow, Glasgow, United Kingdom; 7 Cardiovascular Research Institute Maastricht (CARIM), Maastricht University, Maastricht, The Netherlands; Scuola Superiore Sant'Anna, ITALY

## Abstract

**Objectives:**

Heart transplant (HTx) recipients have a high heart rate (HR), because of graft denervation and are frequently started on β-blockade (BB). We assessed whether BB and HR post HTx are associated with a specific urinary proteomic signature.

**Methods:**

In 336 HTx patients (mean age, 56.8 years; 22.3% women), we analyzed cross-sectional data obtained 7.3 years (median) after HTx. We recorded medication use, measured HR during right heart catheterization, and applied capillary electrophoresis coupled with mass spectrometry to determine the multidimensional urinary classifiers HF1 and HF2 (known to be associated with left ventricular dysfunction), ACSP75 (acute coronary syndrome) and CKD273 (renal dysfunction) and 48 sequenced urinary peptides revealing the parental proteins.

**Results:**

In adjusted analyses, HF1, HF2 and CKD273 (p ≤ 0.024) were higher in BB users than non-users with a similar trend for ACSP75 (p = 0.06). Patients started on BB within 1 year after HTx and non-users had similar HF1 and HF2 levels (p ≥ 0.098), whereas starting BB later was associated with higher HF1 and HF2 compared with non-users (p ≤ 0.014). There were no differences in the urinary biomarkers (p ≥ 0.27) according to HR. BB use was associated with higher urinary levels of collagen II and III fragments and non-use with higher levels of collagen I fragments.

**Conclusions:**

BB use, but not HR, is associated with a urinary proteomic signature that is usually associated with worse outcome, because unhealthier conditions probably lead to initiation of BB. Starting BB early after HTx surgery might be beneficial.

## Introduction

Heart transplantation (HTx) is the treatment of choice for a highly selected group of terminally ill heart failure patients with severe symptoms not responding to optimal medical combined with device-based therapy [1]. Denervation of the graft explains why HTx recipients commonly have a high heart rate, which is an independent predictor of mortality [2–4]. The current study builds on previous observations in a single-center cohort of HTx patients [5,6]. A higher heart rate 3 months after surgery and non-use of β-blockers were associated with increased mortality [5]. In the same cohort [6], elevated right heart pressures were associated with increased urinary levels of the multidimensional urinary classifier HF2 [7]. In line with a position paper of the American Heart Association supporting the use of omics technologies in research on cardiovascular disease [8], the aim of our current study was to explore whether the use of β-blockers or tachycardia after HTx were associated with a specific urinary peptidomic signature. We studied the multidimensional urinary classifiers HF1 [9,10], HF2 [7], ACSP75 [11] and CKD273 [12,13], respectively consisting of 85, 671, 75 and 273 peptide fragments, mainly dysregulated peptide fragments. These markers were developed for the diagnosis of asymptomatic diastolic left ventricular dysfunction [9,10], symptomatic heart failure [7], the prediction of acute coronary events [11], and the decline in glomerular filtration rate [12,13]. We also studied single sequenced urinary peptides, which identify parental proteins and can thereby reveal underlying pathophysiological processes.

## Methods

### Study participants

uPROPHET complies with the Helsinki declaration for research in humans [14]. The study was approved by the Ethics Committee of the University Hospitals Leuven (numbers B322201421186 [S56384] and B322201421045 [S56472]) and passed review by the European Research Council Executive Agency [15]. Recruitment of patients took place at the University Hospitals Leuven in collaboration with the heart transplantation team. All HTx recipients in regular follow-up at the University Hospitals Leuven gave written informed consent and provided a 5-mL mid-morning urine sample for urinary peptidomic analysis. Of the 368 patients enrolled by the end of 2016, 336 had their heart rate measured during right cardiac catheterization within 6 months of the urine sampling and were included in the present analysis.

### Collection of clinical data

A detailed description of the construction and contents of the uPROPHET database is available elsewhere [15]. All potentially relevant clinical information, including anthropometrics, previous medical history, biochemical measurements and use of immunosuppressive, antihypertensive, lipid-lowering and antidiabetic drugs was retrieved from the computerized information system of the University Hospitals Leuven. Hypertension was an office blood pressure of at least 140 mmHg systolic or 90 mmHg diastolic or use of antihypertensive drugs. Right heart hemodynamic measurements included mean right atrial pressure (mRAP), mean pulmonary arterial pressure (mPAP) and mean pulmonary capillary wedge pressure (mPCWP). The right heart pressures were averaged over the respiratory cycle. We applied the 75th percentiles of mRAP (≥10 mm Hg), mPAP (≥24 mm Hg) or mPCWP (≥17 mm Hg) to define elevated right heart pressure.

Venous blood samples were drawn within one week of urine sampling after at least 8 hours of fasting. We measured the concentration of creatinine in serum, using Jaffe’s method [16] with modifications described elsewhere [17] and isotope-dilution mass spectrometry for calibration. We estimated glomerular filtration rate (eGFR) from serum creatinine by the Chronic Kidney Disease Epidemiology Collaboration (CKD-EPI) equation [18]. Diabetes mellitus was a hospital diagnosis, a fasting plasma glucose of 126 mg/dl or higher, or use of antidiabetic agents [19].

### Urinary proteomics

Methods for urine sample preparation, proteome analysis by capillary electrophoresis coupled to mass spectrometry (CE-MS), data processing and sequencing have been published before [15,20–22]. Peptide fragments were combined into a single summary variable, using the MosaCluster software, version 1.7.0 [23]. The so derived multidimensional urinary classifiers HF1 [7,9], HF2 [9], ACSP75 [11] and CKD273 [12,13] are specifically associated with or predictive of asymptomatic diastolic left ventricular dysfunction, advanced heart failure, acute coronary syndrome or deteriorating renal function, and respectively consist of 85, 671, 75 and 273 urinary peptide fragments. They are normally distributed, higher values being associated with worse outcomes. For in-depth analysis of individual single peptides, we selected 48 peptides (S1 Table), which had a detectable signal in over 95% of participants.

### Statistical analysis

For database management and statistical analysis, we used the SAS system, version 9.4 (SAS Institute Inc., Cary, NC). Means were compared using the large-sample z-test or ANOVA and proportions by Fisher’s exact test. We rank normalized the distributions of the urinary peptides by sorting measurements from the smallest to the highest and then applying the inverse cumulative normal function [24]. We adjusted the analyses of the multidimensional classifiers for covariables, including time since transplantation, age, mean arterial pressure, body mass index, total-to-high density lipoprotein (HDL) cholesterol ratio, and the presence of diabetes mellitus. Analyses of HF1, HF2 and ACSP75 were additionally adjusted for eGFR. In the last step of our analyses, we applied partial least squares discriminant analysis (PLS-DA), which is a statistical technique that constructs models for a categorical outcome in relation to correlated high-dimensional explanatory variables [25]. In our study, PLS-DA analysis allowed identifying a set of independent latent factors that were linear combinations of the urinary peptides and that maximized the covariance between use or non-use of β-blockers and the urinary peptides. The importance of each urinary peptide in the construction of the PLS-DA factors was assessed from the Variable Importance in Projection (VIP) score of Wold with the threshold set at 1.1.

## Results

### Patient characteristics

**Fig 1:**
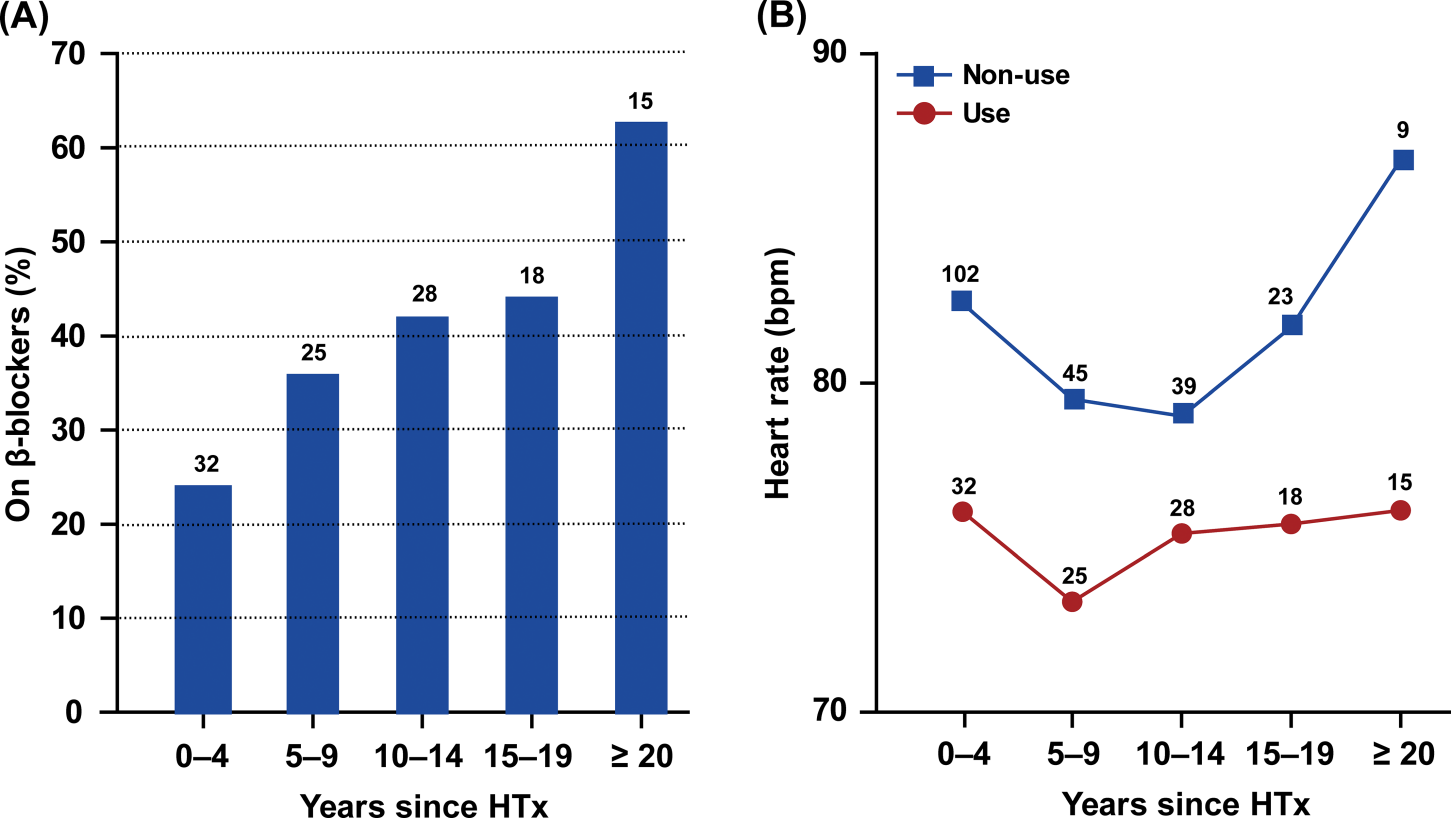
β-Blockers use (A) and heart rate during right heart catheterization (B) by years since heart transplantation. The number of patients contributing to each statistic is given alongside the columns (A) or plotted points (B).

Among the 336 study participants, the causes of end-stage heart failure were ischemic cardiomyopathy in 128 (38.1%), dilated cardiomyopathy in 138 (41.1%) and other etiologies in 70 (20.8%). The urine sample for proteomics was obtained at a median interval of 7.3 years (interquartile range, 2.3 to 13.8 years) after HTx. The 336 patients underwent surgery from August 1988 until October 2016 and included 75 women (22.3%). Mean (±SD) age of the donors was 36.5 ± 13.3 years. In total, 118 patients had been started on β-blockers. The indication to start β-blockade was systemic arterial hypertension in 68 (57.6%) patients, supraventricular tachycardia in 29 (24.6%), ventricular arrhythmia in 6 (5.1%), tremor in 4 (3.4%), sinus tachycardia in 3 (2.5%), migraine in 3 (2.5%), angina pectoris in 2 (1.7%), heart failure post myocardial infarction in 1 (0.1%), hyperthyroidism in 1 (0.1%) and not documented in 1 (0.1%). Fig 1 shows the use of β-blockers and heart rate at the time of urine collection by the time interval since HTx. As shown in S2 Table, patients started on β-blockade within 1 year of HTx (n = 54; 45.8%), compared with those in whom β-blockade was started later (n = 64; 54.2%), were younger (57.1 *vs*. 64.6 years), had lower mRAP (8.4 *vs*. 9.7 mm Hg) and mPAP (21.7 vs. 23.4 mm Hg) and lower serum creatinine (1.52 *vs*. 1.76 mg/dl) and higher eGFR (53.7 *vs*. 43.9 ml/min/1.73 m2).

Table 1 lists the characteristics of the patients dichotomized by β-blocker use or the 75th percentile of heart rate during right heart catheterization (86 beats per minute). Patients on β-blockers compared with non-users, had a higher frequency of hypertension and diabetes mellitus, received their HTx a longer time ago, were older, and had higher body mass index, mPAP, E/e’ ratio, plasma glucose, serum creatinine, but lower heart rate, serum total cholesterol, serum HDL cholesterol and eGFR. Patients with heart rate of 86 beats per minute or higher compared with the lower three fourths of the distribution, were leaner and had lower mRAP, mPCWP and E/A ratio (Table 1). Among users of β-blockers (S3 Table), patient characteristics did not differ between those on cardioselective agents (bisoprolol, celiprolol, metoprolol and nebivolol) and non-cardioselective drugs (carvedilol, propranolol and sotalol). Hypertensive compared with normotensive patients were more obese, had higher serum creatinine and lower eGFR, but had a similar heart rate (S4 Table).

**Table 1:** Baseline characteristics of participants by β-blocker use or heart rate during right heart catheterization.

Characteristic	Use	Non-use	<86 beats/minute	≥86 beats/minute
Number of participants (%)	118	218	241	95
Women	23 (19.5)	52 (23.9)	54 (22.4)	21 (22.1)
Hypertension	108 (91.5)	179 (82.1)*	205 (85.1)	82 (86.3)
Diabetes mellitus	37 (31.4)	46 (21.1)*	59 (24.5)	24 (25.3)
Ischemic cardiomyopathy	52 (44.1)	76 (34.9)	96 (39.8)	32 (33.7)
Dilated cardiomyopathy	48 (40.7)	90 (41.3)	95 (39.4)	43 (45.3)
Elevated right heart pressure	63 (53.4)	97 (44.5)	122 (50.6)	38 (40.0)
Mean (± SD) of characteristic				
Years since HTx	10.7 (4.5–15.5)	5.4 (0.9–12.0)§	7.3 (2.7–13.5)	7.3 (0.7–14.3)
Age (years)	61.3 ± 12.0	54.4 ± 15.4§	57.3 ± 13.9	55.6 ± 16.3
Body mass index (kg/m2)	26.1 ± 4.9	24.7 ± 3.8†	25.5 ± 4.4	24.5 ± 3.6*
Systolic pressure (mm Hg)	144.1 ± 23.9	141.0 ± 19.4	142.6 ± 20.7	140.9 ± 22.2
Diastolic pressure (mm Hg)	85.7 ± 12.7	84.7 ± 10.4	85.0 ± 11.2	85.2 ± 11.4
Heart rate (beats per minute)	75.3 ± 10.4	81.3 ± 12.5§	73.5 ± 8.3	93.6 ± 7.5§
Office heart rate (beats per minute)	77.3 ± 11.9	80.7 ± 12.5†	75.6 ± 10.7	89.4 ± 10.7§
mRAP (mm Hg)	9.1 ± 3.2	8.6 ± 2.8	9.0 ± 2.9	8.2 ± 3.1*
mPAP (mm Hg)	22.6 ± 4.7	21.3 ± 4.6*	22.0 ± 4.7	21.1 ± 4.6
mPCWP (mm Hg)	15.1 ± 4.5	14.1 ± 4.0	14.9 ± 4.2	13.3 ± 3.9†
Ejection fraction (%)	59.4 ± 2.4	58.9 ± 4.6	59.2 ± 4.3	58.8 ± 2.9
E/A ratio	2.28 ± 1.40	2.09 ± 1.30	2.27 ± 1.51	1.86 ± 0.67†
E/e’ ratio	6.89 ± 2.74	6.19 ± 2.07*	6.58 ± 2.42	6.09 ± 2.13
Serum total cholesterol (mg/dl)	149.7 ± 34.3	159.3 ± 34.7*	156.3 ± 34.2	155.0 ± 36.7
Serum HDL cholesterol (mg/dl)	53.8 ± 16.7	59.3 ± 17.1†	57.3 ± 17.4	57.6 ± 16.6
Plasma glucose (mg/dl)	106.1 ± 28.5	98.3 ± 20.8†	101.4 ± 24.5	100.0 ± 23.0
Serum creatinine (mg/dl)	1.65 ± 0.51	1.30 ± 0.43§	1.44 ± 0.48	1.37 ± 0.51
eGFR (ml/min/1.73 m2)	48.4 ± 22.2	65.7 ± 25.1§	57.8 ± 23.5	64.2 ± 29.6

Abbreviations: mRAP, mean right atrial pressure; mPAP, mean pulmonary arterial pressure; mPCWP, mean pulmonary capillary wedge pressure; HDL, high-density lipoprotein; eGFR, glomerular filtration rate estimated from serum creatinine. Heart rate refers to the heart rate measured during right heart catheterization. A heart rate of 86 during right heart catheterization corresponded to the 75th percentile of the distribution. Office heart rate was measured on the day of the urine collection within 6 months of the catheterization. For years since transplantation the median (interquartile range) is given. Hypertension was an office blood pressure of at least 140 mmHg systolic or 90 mmHg diastolic or use of antihypertensive drugs. Diabetes mellitus was a hospital diagnosis, a fasting plasma glucose of 126 mg/dl or higher, or use of antidiabetic agents. Elevated right heart pressure was mRAP (≥10 mm Hg), mPAP (≥24 mm Hg), or mPCWP (≥17 mm Hg) equal to or exceeding the 75th percentile of the distributions.

Significance of the between-group difference

* p ≤ 0.05

† p ≤ 0.01

§ p ≤ 0.0001.

### Use of medications

Of 336 patients, 67 (19.9%) were taking cyclosporine, 260 (77.4%) tacrolimus, 267 (79.5%) mycophenolate mofetil, 20 (6.0%) azathioprine, 19 (5.7%) everolimus and 122 (36.3%) methylprednisolone. Sixteen (4.8%) were taking a single immunosuppressant, 221 (65.8%) two drugs and 99 (29.5%) three drugs. The most common combination was tacrolimus plus mycophenolate mofetil (209 patients; 62.2%). The number of patients taking one or more blood pressure lowering drugs was 240 (71.4%), of whom 66 (27.5%) were on a thiazide or a loop diuretic, 118 (35.1%) on β-blockers, 148 (61.7%) on angiotensin-converting enzyme inhibitors or angiotensin I receptor blockers, 99 (41.3%) on calcium-channel blockers and 20 (8.3%) on aldosterone antagonists. The number of patients on antidiabetic treatment totaled 88, of whom 53 (60.2%) were on oral therapy and 35 (39.8%) on insulin.

β-Blockers users *vs*. non-users (Table 2) were less frequently taking tacrolimus (67.8% *vs*. 82.6%), but were more frequently treated with cyclosporine (29.7% *vs*. 14.7%), thiazides (17.8% *vs*. 6.4%), inhibitors of the renin-angiotensin system (54.2% *vs*. 38.5%) and insulin (16.1% vs. 7.3%). The differences in medication use between patients with fast *vs*. slow heart rate were all nonsignificant except for the use of β-blockers (Table 2).

**Table 2:** Use of medications by β-blocker use or heart rate during right heart catheterization.

Characteristic	Use	Non-use	<86 beats/minute	≥86 beats/minute
Number of participants (%)	118	218	241	95
Immunosuppressive treatment				
Calcineurin inhibitor	115 (97.5)	212 (97.3)	233 (96.7)	94 (99.0)
Tacrolimus	80 (67.8)	180 (82.6)†	189 (78.4)	71 (74.7)
Cyclosporine	35 (29.7)	32 (14.7)‡	44 (18.3)	23 (24.2)
Antiproliferative agents	98 (83.1)	189 (86.7)	205 (85.1)	82 (86.3)
mTOR inhibitors	8 (6.8)	11 (5.1)	12 (5.0)	7 (7.4)
Methylprednisolone	42 (35.6)	80 (36.7)	82 (34.0)	40 (42.1)
Antihypertensive drugs				
Any drug class	100 (84.8)	140 (64.2)§	173 (71.8)	67 (70.5)
β-blockers	118 (100)	0 (0) …	95 (39.4)	23 (24.2)†
Thiazides	21 (17.8)	14 (6.4)‡	29 (12.0)	6 (6.3)
Loop diuretics	10 (8.5)	24 (11.0)	25 (10.4)	9 (9.5)
Aldosterone antagonists	4 (3.4)	16 (7.3)	14 (5.8)	6 (6.3)
Calcium channel blockers	31 (26.3)	68 (31.2)	70 (29.1)	29 (30.5)
RAS inhibitors	64 (54.2)	84 (38.5)†	110 (45.6)	38 (40.0)
Use of statins	112 (94.9)	202 (92.7)	222 (92.1)	92 (96.8)
Use of antidiabetic drugs				
Insulin	19 (16.1)	16 (7.3)*	25 (10.4)	10 (10.5)
Other agents	22 (18.6)	31 (14.2)	39 (16.2)	14 (14.7)

Abbreviations: mTOR, mammalian target of rapamycin; RAS renin-angiotensin system. Drugs by class: calcineurin inhibitors, tacrolimus and cyclosporine; antiproliferative agents, azathioprine and mycophenolate mofetil; mTOR inhibitors, everolimus and sirolimus; RAS inhibitors, converting-enzyme inhibitors and angiotensin II type-1 receptor blockers. A heart rate of 86 during right heart catheterization corresponded to the 75th percentile of the distribution.

Significance of the between-group differences: …

p not computed

* p ≤ 0.05

† p ≤ 0.01

‡ p ≤ 0.001

§ p ≤ 0.0001.

### Multidimensional classifiers

We adjusted the analyses for time since transplantation, age, mean arterial pressure, body mass index, total-to-HDL cholesterol ratio, and the presence of diabetes mellitus. Analyses of HF1, HF2 and ACSP75 were additionally adjusted for eGFR. With such adjustments applied (Table 3), levels of HF1, HF2 and CKD273 were significantly (p ≤ 0.024) higher in β-blockers users compared with non-users with a similar trend for ACSP75 (p = 0.060). In contrast, there were no differences in the levels of these urinary classifiers (p ≥ 0.27) by category of heart rate (Table 3). In similarly adjusted models including both β-blocker use vs. non-use and heart rate category, all four classifiers were higher in β-blocker users compared with non-users (p ≤ 0.045) with no differences (p ≥ 0.16) between the heart rate categories (Table 3). Sensitivity analyses additionally adjusted for elevated right heart pressure (0,1), the use of antihypertensive drugs other than β-blockers (0,1) and immunosuppressants (by drug class) were confirmatory.

**Table 3:** Urinary levels of classifiers by β-blocker use or heart rate during right heart catheterization.

Model Classifier	β-blocker use *vs*. non-use				Heart rate categories			
	Use	Non-use	Δ (95% CI)	*p*	<86 bpm	≥86 bpm	Δ (95% CI)	*p*
Adjusted								
HF1	–0.53 ± 0.09	–0.78 ± 0.06	–0.25 (–0.46, –0.03)	0.024	–0.68 ± 0.06	–0.72 ± 0.09	0.04 (–0.18, 0.25)	0.73
HF2	0.07 ± 0.06	–0.12 ± 0.04	–0.19 (–0.34, –0.05)	0.009	–0.08 ± 0.04	0.00 ± 0.06	–0.08 (–0.23, 0.06)	0.27
ACSP75	0.47 ± 0.24	–0.11 ± 0.18	–0.59 (–1.20, 0.02)	0.060	0.00 ± 0.16	0.33 ± 0.26	–0.33 (–0.94, 0.28)	0.29
CKD273	0.19 ± 0.04	0.07 ± 0.03	–0.12 (–0.21, –0.02)	0.017	0.12 ± 0.03	0.07 ± 0.04	0.05 (–0.05, 0.15)	0.31
Mutually adjusted								
HF1	–0.53 ± 0.09	–0.78 ± 0.06	–0.24 (–0.46, –0.03)	0.026	–0.65 ± 0.06	–0.66 ± 0.09	0.01 (–0.20, 0.23)	0.92
HF2	0.10 ± 0.06	–0.10 ± 0.04	–0.20 (–0.35, –0.06)	0.006	–0.05 ± 0.04	0.05 ± 0.06	–0.10 (–0.25, 0.04)	0.16
ACSP75	0.58 ± 0.26	–0.04 ± 0.18	–0.63 (–1.24, –0.01)	0.045	0.07 ± 0.17	0.47 ± 0.27	–0.40 (–1.01, 0.21)	0.20
CKD273	0.17 ± 0.04	0.06 ± 0.03	–0.11 (–0.21, –0.02)	0.023	0.14 ± 0.03	0.10 ± 0.04	0.04 (–0.06, 0.13)	0.47

Values are mean ± SE or mean between-group differences (Δ) with 95% confidence interval (95% CI). All models were adjusted for time since transplantation, age, mean arterial pressure, body mass index, total-to-HDL cholesterol ratio and the presence of diabetes mellitus. For HF1, HF2 and ACSP75, models were additionally adjusted for glomerular filtration rate estimated from serum creatinine. Mutually adjusted models included both β-blocker use *vs*. non-use and heart rate during right heart catheterization categorized by 86 beats per minute (bpm), the 75th percentile of the distribution.

**Fig 2:**
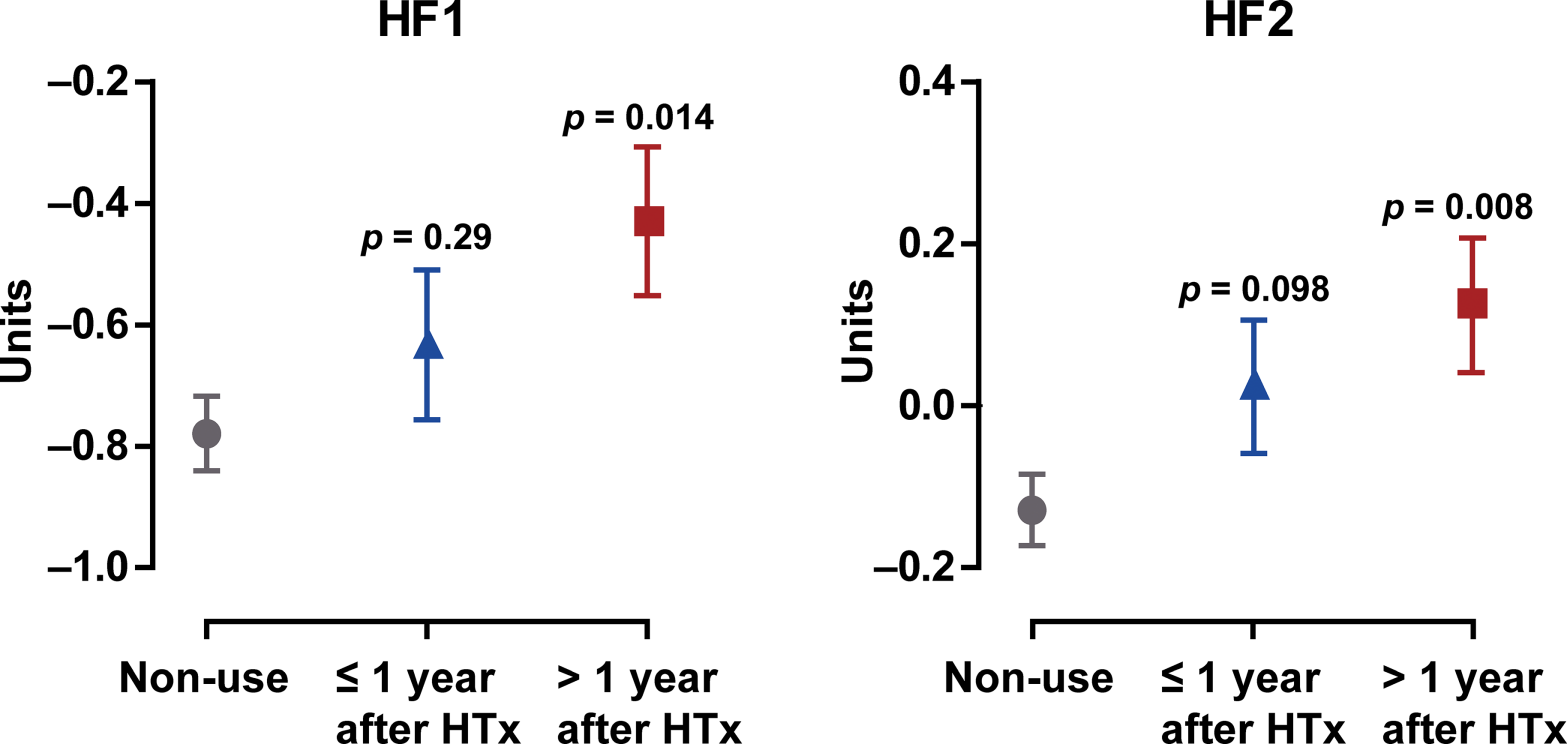
Levels of the urinary classifiers HF1 and HF2 in non-users of β-blockers and in users started on β-blockade within 1 year of heart transplantation (n = 54) or later (n = 64). Estimates given with SE were adjusted for time since transplantation, age, mean arterial pressure, body mass index, total-to-HDL cholesterol ratio, glomerular filtration rate estimated from serum creatinine and the presence of diabetes mellitus. p values denote the significance of the difference between non-users and users.

β-Blockade was initiated at a median interval of 1.7 years (interquartile range, 0.3 to 6.0 years) after HTx. For HF1 and HF2, but not for ACSP75 and CKD273, there was a time trend associated with early (within 1 year after HTx; n = 54) vs. later (n = 64) start of β-blockade (Fig 2). The indications for starting β-blockade were similar (p = 0.58) in patients started early *vs*. later.

### Sequenced urinary peptides

**Fig 3:**
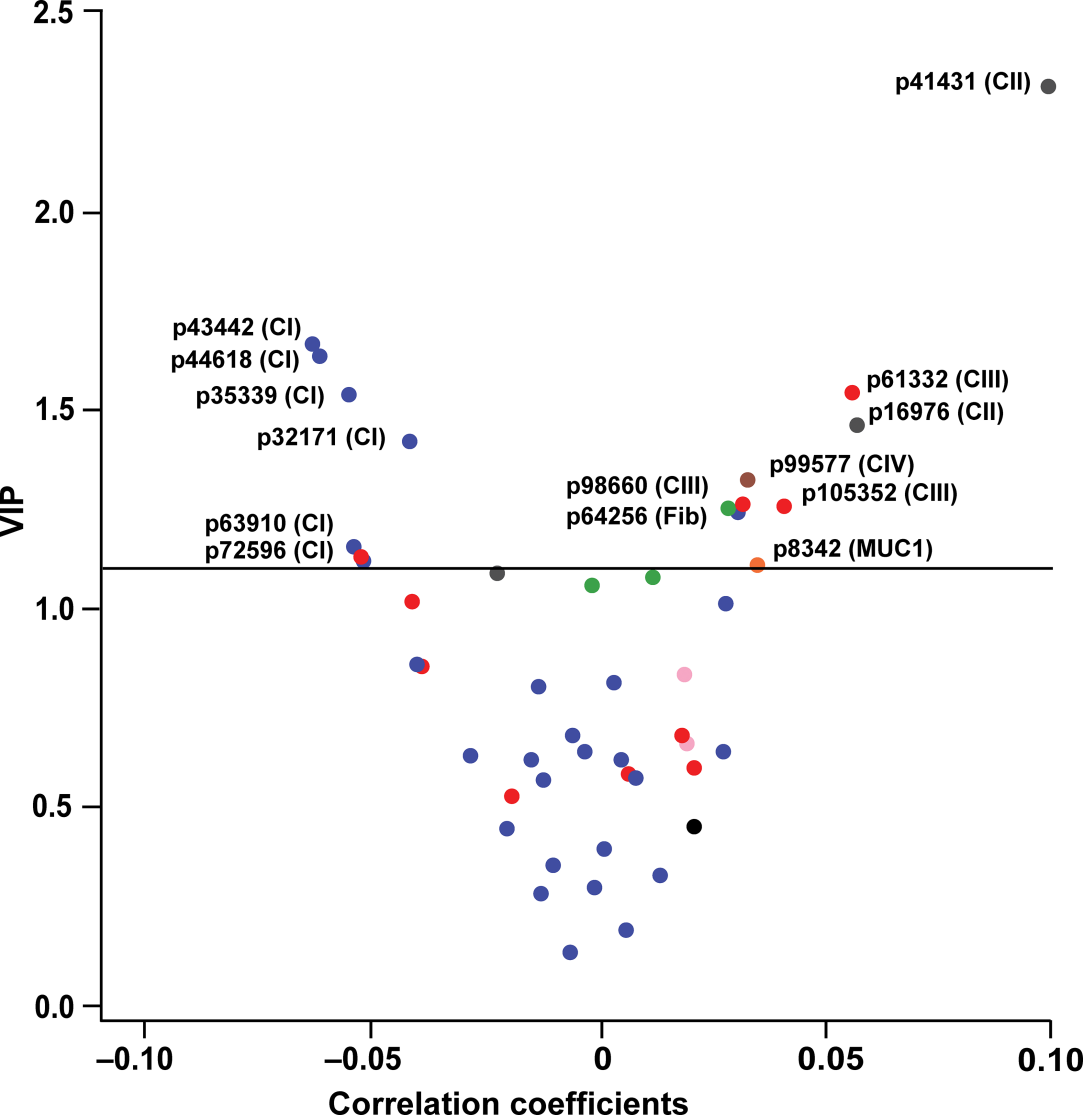
V-plots generated by partial least squares discriminant analysis. Variable Importance in Projection (VIP) scores indicate the importance of each urinary fragment in the construction of the partial least squares factors and are plotted against the centered and rescaled correlation coefficients. These correlation coefficients reflect the associations of β-blockers use *vs*. non-use with the urinary fragments. The urinary peptides associated with non-use (left side of the V plot) included collagen I fragments. The urinary peptides associated with use of β-blockers included fragments of collagen II and III and a fragment of collagen IV, the fibrinogen α chain and the mucin-1 subunit α. Colors identify fragments derived from collagen I (blue), II (grey), III (red), IV (brown), V (pink), mucin-1 subunit α (orange), fibrinogen (green), and uromodulin (black).

The PLS-DA analyses yielded two latent factors that accounted for 26.9% of the overall variance in the urinary peptides and 18.4% of the variance in use *vs*. non-use of β-blockers. Fig 3 depicts the PLS-DA derived Variance in Projection (VIP) scores *vs*. the centered and rescaled correlation coefficients. VIP scores indicate the importance of each urinary fragment in the construction of the partial least square factors. The correlation coefficients reflect the associations of β-blocker use *vs*. non-use with the urinary fragments. The urinary peptides associated with non-use of β-blockers (left side of the V plot) included collagen I fragments (p32171, p35339, p43442, p44618, p63910 and p72596). The urinary peptides associated with use of β-blockers included fragments of collagen II (p16976 and p41431) and III (p61332, p98660 and P105352) and a fragment of collagen IV (p99577), the fibrinogen α chain (p64256) and the mucin-1 subunit α (p8342).

### Sensitivity analyses

The correlation between heart rate during right heart catheterization and office heart rate at the time of the urine collection was 0.68 (p < 0.0001). Replacing heart rate during right heart catheterization by office heart rate therefore produced consistent results (S5–S7 Tables).

We ran an additional analysis dictomized according to whether or not patients were on inhibitors of the renin-angiotensin system. As before, we adjusted these analyses for time since transplantation, age, heart rate, mean arterial pressure, body mass index, total-to-HDL cholesterol ratio, and the presence of diabetes mellitus. The analyses of HF1, HF2 and ACSP75 were additionally adjusted for eGFR. With these adjustments applied, levels of HF1, HF2, ACSP75 and CKD273 were significantly (p ≤ 0.046) higher in users of inhibitors of the renin-angiotensin system compared with non-users (S8 Table).

## Discussion

The key finding of our manuscript was that use *vs*. non-use of β-blockers was associated with specific urinary proteomic signatures, whereas heart rate was not (Table 3). β-blocker use correlated with higher levels of the multidimensional classifiers HF1, HF2, ACSP75 and CKD273, which in other studies were indicative of a worse haemodynamic condition [7] or predicted cardiovascular [26], cardiac [26] or coronary [11] events or decline in renal function [12,27]. Along similar lines, β-blockade was associated with higher levels of various fragments of collagen II and III and a fragment of the fibrinogen α chain and the mucin-1 subunit **α**, which are indicative of diastolic left ventricular dysfunction [28] or renal impairment [13,27]. These observations probably reflect reverse causality, indicating that unhealthier HTx patients, as reflected by their urinary biomarkers, were more likely to be started on a β-blocker. In keeping with this idea, patients on β-blockers compared with non-users, had a higher frequency of hypertension and diabetes mellitus, were older and more obese, and had lower eGFR (Table 1).

For HF1 and HF2 there was a time trend associated with the start of β-blockade (Fig 2), which were adjusted for confounders as patients in whom beta blockers were initiated within one year had favorable risk profile shown in S2 Table. Patients started on β-blockers early after HTx had HF1 and HF2 levels not different from those in non-users, whereas in patients started on β-blockers 1 year after HTx HF1 and HF2 levels were significantly higher than in non-users. These observations are in line with our previous report [5], in which all-cause mortality was studied in relation to heart rate during right heart catheterization and use of β-blockers at 3 months after HTx. The study included 461 HTx patients with a mean follow-up of 11.9 years. Patients receiving β-blockers had lower mortality than those who did not use β-blockers [5]. In multivariable-adjusted analyses, older age, longer hospitalization, higher mPAP, higher heart rate at 3 months (hazard ratio [HR], 1.22 per 10 beats per minute; p = 0.02) and use *vs*. non-use of β-blockers (HR, 1.43; p = 0.05) predicted higher mortality [5]. Survival function analysis demonstrated that β-blockade delayed death up to 15 years after transplantation (p = 0.04), whereafter the survival curves between user and non-users of β-blockers coincided. To our knowledge, no other study described the early protective effect of β-blockade in HTx patients. Our current observations (Fig 2) and this report [5] suggest that starting β-blockade early in HTx patients might be protective over and beyond lowering of heart rate, a hypothesis worth testing in a randomized clinical trial.

High heart rate is an independent predictor of mortality both in the general population [29] and HTx recipients [2–4]. Whether or not β-blockade confers benefit in patients with cardiovascular disease remains controversial [30–32]. In the Reduction of Atherothrombosis for Continued Health (REACH) registry, propensity score matching was used for the primary analyses involving 21,860 patients with a median follow-up of 44 months. Event rates were not significantly different in patients on β-blockers compared with those not on β-blockers for any of the outcomes tested, even in the prior myocardial infarction cohort (HR, 0.90; p = 0.14) [31]. A meta-analysis [32] including 17,397 patients with coronary heart disease without previous myocardial infarction or reduced ejection fraction, showed that β-blocker use did not entail any reduction in all-cause mortality (odds ratio [OR] 0.91; p = 0.16) or cardiac mortality (OR, 0.93; p = 0.41). However, double-blind placebo-controlled trials of β-blocker use after myocardial infarction published more than 30 year ago [30], in contrast to the more recent observational studies [31,32], demonstrated substantial benefit of β-blockade in secondary prevention.

In an earlier published population study (n = 782), a 1-SD increment in a urinary collagen I fragment was associated with 0.183 cm/s (p = 0.025) lower e’ (peak velocity of the mitral annular movement in early diastole) and 0.210 cm/s (p = 0.0012) greater E/e' (peak velocity of the transmitral blood flow in early diastole divided by e’) [28]. E/e' decreased by 0.168 (p = 0.018) in relation to a urinary fragment of collagen III [28]. Lower e’ and higher E/e’ reflect worse diastolic left ventricular function. In heart failure patients, β-blockers inhibit the myocardial degradation of collagen [33,34], possibly via a transforming growth factor β1-mediated mechanism [33]. These observations [33,34], and the differences in the hemodynamic conditions leading to the initiation of β-blockade (Table 1) might partially explain why in our current study β-blockade was associated with higher urinary levels of collagen III fragments and non-use with higher levels of collagen I (Fig 3).

Our study must be interpreted within the context of its limitations. First, our analysis was cross-sectional. However, we ascertained that over the interval from initiation of β-blockade until the assessment of the urinary proteome all 118 patients started on a β-blocker continued their treatment. Second, our study was a single-center study so that the generalizability of our observations remains to be confirmed in other HTx cohorts. Third, the observational nature of our findings does not allow making causal interferences. Finally, although we adjusted for known confounders, prescription of any drug, including β-blockers during follow-up of heart transplant recipients, is necessarily biased, which may confound the results to an extent that cannot be fully appreciated. However, the sensitivity analysis of inhibitors of the renin-angiotensin system supported our hypothesis that reverse causality explained the association between β-blockade and the urinary biomarkers. Unfortunately, we did not collect urine samples prior to the initiation of β-blockade or inhibitors of the renin angiotensin system, so that we could not assess the changes in the urinary biomarker profile induced by these drugs.

In conclusion, in HTx recipients, use of β-blockers *vs*. non-use, but not heart rate, is associated with a specific urinary proteomic signature that includes multidimensional classifiers and urinary fragments of collagen and other proteins, usually associated with adverse health outcomes [7,11,12,26–28]. Reverse causality probably explains these findings. The urinary levels of the multidimensional classifiers HF1 and HF2 reportedly associated with worse left ventricular function in the general population [7] or increased right heart pressures in HTx patients [6], increased with later post-surgery initiation of β-blockade. This finding along with our previous report [5], suggest that there might be benefit in starting a β-blocker soon after surgery in all HTx patients, but this hypothesis remains to be confirmed in a randomized clinical trial.

## Supporting information

S1 Table Urinary peptide fragments with known amino acid sequence. (DOC)

S2 Table Baseline characteristics of participants by starting year of β-blockade. (DOC)

S3 Table Baseline characteristics of participants by type of β-blockers. (DOC)

S4 Table Baseline characteristics of participants by blood pressure category. (DOC)

S5 Table Baseline characteristics of participants by β-blocker use or office heart rate. (DOCX)

S6 Table Use of medications by β-blocker use or office heart rate. (DOCX)

S7 Table Urinary levels of classifiers by β-blocker use or office heart rate. (DOCX)

S8 Table Urinary levels of classifiers by RAS inhibitor use. (DOCX)
