# Supplementary material for: Urinary proteomic signatures associated with β-blockade and heart rate in heart transplant recipients
Source: PLoS One. 2018 Sep 24;13(9):e0204439. doi: 10.1371/journal.pone.0204439 (PMC6152976; doi:10.1371/journal.pone.0204439)
Supplement: S1 Table — (DOC) [file pone.0204439.s001.doc]

**S1 Table.**

**Urinary peptide fragments with known amino acid sequence (starts)**

| **ID** | **Sequence** | **Protein** |
| --- | --- | --- |
| p8342 | TTLASHSTK | Mucin-1 subunit alpha |
| p16976 | DGpSGAEGpPGp | Collagen alpha-1 (II) chain |
| p24117 | SpGPDGKTGpPGP | Collagen alpha-1 (I) chain |
| p27517 | ApGEDGRpGPpGP | Collagen alpha-1 (II) chain |
| p32171 | ApGDRGEpGPpGPA | Collagen alpha-1 (I) chain |
| p34766 | PpGPpGPpGpPGPPS | Collagen alpha-1 (I) chain |
| p35339 | ApGDRGEpGPpGPAG | Collagen alpha-1 (I) chain |
| p40243 | SpGSpGPDGKTGPPGp | Collagen alpha-1 (I) chain |
| p41431 | GPpGKpGDDGEAGKPG | Collagen alpha-1 (II) chain |
| p41601 | DGQPGAKGEpGDAGAK | Collagen alpha-1 (I) chain |
| p43442 | VGPpGPPGPpGPpGPPS | Collagen alpha-1 (I) chain |
| p44618 | VGPpGPpGPpGpPGPPS | Collagen alpha-1 (I) chain |
| p45445 | GpEGPpGEPGpPGPPGP | Collagen alpha-2 (V) chain |
| p46880 | GSEADHEGTHSTKRG | Fibrinogen alpha chain |
| p48106 | SpGSpGPDGKTGPPGpAG | Collagen alpha-1 (I) chain |
| p49303 | VGPpGPpGPpGPpGPPSA | Collagen alpha-1 (I) chain |
| p50840 | DGApGKNGERGGpGGpGP | Collagen alpha-1 (III) chain |
| p53035 | VGpPGpPGPpGPpGPPSAG | Collagen alpha-1 (I) chain |
| p57537 | NDGApGKNGERGGpGGpGP | Collagen alpha-1 (III) chain |
| p61332 | ApGAPGGKGDAGApGERGpPG | Collagen alpha-1 (III) chain |
| p61573 | DEAGSEADHEGTHSTKR | Fibrinogen alpha chain |
| p63910 | DDGEAGKPGRpGERGpPGP | Collagen alpha-1 (I) chain |
| p64256 | DEAGSEADHEGTHSTKRG | Fibrinogen alpha chain |
| p65746 | SGSVIDQSRVLNLGPITR | Uromodulin |

**S1 Table.**

**Urinary peptide fragments with known amino-acid sequence (continued)**

| **ID** | **Sequence** | **Protein** |
| --- | --- | --- |
| p70674 | EGSpGRDGSpGAKGDRGETGP | Collagen alpha-1 (I) chain |
| p72596 | NGDDGEAGKPGRPGERGpPGp | Collagen alpha-1 (I) chain |
| p73246 | NGDDGEAGKPGRpGERGppGP | Collagen alpha-1 (I) chain |
| p74065 | DAGApGApGGKGDAGApGERGpPG | Collagen alpha-1 (III) chain |
| p77763 | DGQpGAKGEpGDAGAKGDAGPpGP | Collagen alpha-1 (I) chain |
| p77952 | VGEpGPAGSKGESGNKGEpGSAGP | Collagen alpha-2 (I) chain |
| p78332 | AGPpGEAGKpGEQGVPGDLGApGP | Collagen alpha-1 (I) chain |
| p78843 | NSGEpGApGSKGDTGAKGEPGpVG | Collagen alpha-1 (I) chain |
| p79136 | AGPpGEAGKpGEQGVpGDLGApGP | Collagen alpha-1 (I) chain |
| p80891 | ADGQPGAKGEpGDAGAKGDAGPPGp | Collagen alpha-1 (I) chain |
| p81196 | NGApGNDGAKGDAGApGApGSQGApG | Collagen alpha-1 (I) chain |
| p81758 | ADGQpGAKGEpGDAGAKGDAGPPGp | Collagen alpha-1 (I) chain |
| p84542 | QNGEpGGKGERGApGEKGEGGPpG | Collagen alpha-1 (III) chain |
| p85761 | ADGQpGAKGEpGDAGAKGDAGPpGPA | Collagen alpha-1 (I) chain |
| p89233 | KGNSGEpGApGSKGDTGAKGEpGPVG | Collagen alpha-1 (I) chain |
| p98596 | ApGPAGSRGApGPQGpRGDKGETGERG | Collagen alpha-1 (III) chain |
| p98660 | GApGQNGEpGGKGERGApGEKGEGGPpG | Collagen alpha-1 (III) chain |
| p99577 | PGRpGLDGERGRPGPAGPpGPpGPSSN | Collagen alpha-6 (IV) chain |
| p104786 | pGMPGADGPPGHPGKEGppGEKGGQGpPG | Collagen alpha-1 (V) chain |
| p105352 | SGHPGSPGSPGYQGPpGEPGQAGPSGPpGP | Collagen alpha-1 (III) chain |
| p111001 | ERGEAGIpGVpGAKGEDGKDGSpGEpGANG | Collagen alpha-1 (III) chain |
| p115491 | ESGREGApGAEGSpGRDGSpGAKGDRGETGP | Collagen alpha-1 (I) chain |
| p118163 | LTGSpGSpGpDGKTGPPGPAGQDGRPGPpGppG | Collagen alpha-1 (I) chain |
| p127031 | ENGKPGEpGpKGDAGApGApGGKGDAGApGERGpPG | Collagen alpha-1 (III) chain |
