# Supplementary material for: Urinary proteomic signatures associated with β-blockade and heart rate in heart transplant recipients
Source: PLoS One. 2018 Sep 24;13(9):e0204439. doi: 10.1371/journal.pone.0204439 (PMC6152976; doi:10.1371/journal.pone.0204439)
Supplement: S3 Table — (DOC) [file pone.0204439.s003.doc]

**S3 Table.**

**Baseline characteristics of participants by type of β-blockade**

| **Characteristic** | **Cardioselective** | **Not  cardioselective** | ***p*** |
| --- | --- | --- | --- |
| Number of participants (%) | 91 | 27 |  |
| Women | 17 (18.7) | 6 (22.2) | 0.68 |
| Hypertension | 82 (90.1) | 26 (96.3) | 0.31 |
| Diabetes mellitus | 28 (30.8) | 9 (33.3) | 0.80 |
| Ischemic cardiomyopathy | 43 (47.3) | 9 (33.3) | 0.20 |
| Dilated cardiomyopathy | 33 (36.3) | 15 (55.6) | 0.073 |
| Elevated right heart pressure | 49 (53.9) | 14 (51.9) | 0.86 |
| Mean (± SD) of characteristic |  |  |  |
| Years since transplantation | 9.3 (4.0–15.5) | 13.2 (6.4–15.7) | 0.51 |
| Age (years) | 60.8 ± 12.5 | 62.7 ± 9.9 | 0.49 |
| Body mass index (kg/m^2^) | 26.0 ± 5.0 | 26.5 ± 4.7 | 0.63 |
| Systolic pressure (mm Hg) | 143.0 ± 24.4 | 147.9 ± 22.4 | 0.36 |
| Diastolic pressure (mm Hg ) | 85.4 ± 12.5 | 86.7 ± 13.6 | 0.66 |
| Heart rate (beats per minute) | 76.0 ± 10.7 | 72.9 ± 9.1 | 0.16 |
| mRAP (mm Hg) | 9.1 ± 3.2 | 9.1 ± 3.1 | 0.98 |
| mPAP (mm Hg) | 22.8 ± 4.9 | 21.8 ± 4.3 | 0.32 |
| mPCWP (mm Hg) | 15.1 ± 4.4 | 15.0 ± 4.8 | 0.96 |
| Serum total cholesterol (mg/dl) | 151.8 ± 35.9 | 142.3 ± 27.7 | 0.21 |
| Serum HDL cholesterol (mg/dl) | 55.0 ± 17.4 | 49.9 ± 13.7 | 0.17 |
| Plasma glucose (mg/dl) | 105.8 ± 30.2 | 106.8 ± 22.6 | 0.88 |
| Serum creatinine (mg/dl) | 1.61 ± 0.52 | 1.78 ± 0.48 | 0.14 |
| eGFR (ml/min/1.73 m^2^) | 50.1 ± 23.2 | 42.4 ± 17.7 | 0.11 |

Abbreviations: mRAP, mean right atrial pressure; mPAP, mean pulmonary arterial pressure; mPCWP, mean pulmonary capillary wedge pressure; HDL, high-density lipoprotein; eGFR, glomerular filtration rate estimated from serum creatinine. For years since transplantation the median (interquartile range) is given. Cardioselective β‑blockers included bisoprolol, celiprolol, metoprolol and nebivolol, and non-cardioselective β‑blockers carvedilol, propranolol and sotalol. Elevated right heart pressure was mRAP (≥10 mm Hg), mPAP (≥24 mm Hg), or mPCWP (≥17 mm Hg) equal to or exceeding the 75th percentile of the distributions. Hypertension was an office blood pressure of at least 140 mmHg systolic or 90 mmHg diastolic or use of antihypertensive drugs. Diabetes mellitus was a hospital diagnosis, a fasting plasma glucose of 126 mg/dl or higher, or use of antidiabetic agents.
