# Supplementary material for: Urinary proteomic signatures associated with β-blockade and heart rate in heart transplant recipients
Source: PLoS One. 2018 Sep 24;13(9):e0204439. doi: 10.1371/journal.pone.0204439 (PMC6152976; doi:10.1371/journal.pone.0204439)
Supplement: S5 Table — (DOCX) [file pone.0204439.s005.docx]

**S5 Table.**

**Baseline characteristics of participants by β-blocker use or office heart rate**

| **Characteristic** | **Use** | **Non-use** | **<88 beats/minute** | **≥88 beats/minute** |
| --- | --- | --- | --- | --- |
| Number of participants (%) | 118 | 218 | 245 | 91 |
| Women | 23 (19.5) | 52 (23.9) | 59 (24.1) | 16 (17.6) |
| Hypertension | 108 (91.5) | 179 (82.1)* | 205 (83.7) | 82 (90.1) |
| Diabetes mellitus | 37 (31.4) | 46 (21.1)* | 54 (22.0) | 29 (31.9) |
| Ischemic cardiomyopathy | 52 (44.1) | 76 (34.9) | 86 (35.1) | 42 (46.2) |
| Dilated cardiomyopathy | 48 (40.7) | 90 (41.3) | 101 (41.2) | 37 (40.7) |
| Elevated right heart pressure | 63 (53.4) | 97 (44.5) | 130 (53.1) | 30 (33.0)‡ |
| Mean (± SD) of characteristic |  |  |  |  |
| Years since HTx | 10.7 (4.5–15.5) | 5.4 (0.9–12.0)§ | 7.5 (3.3–14.1) | 5.5 (0.4–12.8) |
| Age (years) | 61.3 ± 12.0 | 54.4 ± 15.4§ | 56.8 ± 14.3 | 56.8 ± 15.6 |
| Body mass index (kg/m2) | 26.1 ± 4.9 | 24.7 ± 3.8† | 25.2 ± 4.4 | 25.3 ± 3.8 |
| Systolic pressure (mm Hg) | 144.1 ± 23.9 | 141.0 ± 19.4 | 143.0 ± 21.5 | 139.6 ± 19.9 |
| Diastolic pressure (mm Hg ) | 85.7 ± 12.7 | 84.7 ± 10.4 | 85.3 ± 11.6 | 84.4 ± 10.4 |
| Heart rate (beats per minute) | 75.3 ± 10.4 | 81.3 ± 12.5§ | 75.6 ± 10.3 | 88.9 ± 11.4§ |
| Office heart rate (beats per minute) | 77.3 ± 11.9 | 80.7 ± 12.5† | 73.8 ± 8.5 | 94.9 ± 6.7§ |
| mRAP (mm Hg) | 9.1 ± 3.2 | 8.6 ± 2.8 | 9.0 ± 2.9 | 8.3 ± 3.0 |
| mPAP (mm Hg) | 22.6 ± 4.7 | 21.3 ± 4.6* | 22.0 ± 4.6 | 21.2 ± 4.8 |
| mPCWP (mm Hg) | 15.1 ± 4.5 | 14.1 ± 4.0 | 14.8 ± 4.2 | 13.5 ± 3.9† |
| Ejection fraction (%) | 59.4 ± 2.4 | 58.9 ± 4.6 | 59.3 ± 2.5 | 58.6 ± 6.3 |
| E/A ratio | 2.28 ± 1.40 | 2.09 ± 1.30 | 2.29 ± 1.49 | 1.80 ± 0.67§ |
| E/e’ ratio | 6.89 ± 2.74 | 6.19 ± 2.07* | 6.59 ± 2.36 | 6.02 ± 2.27* |
| Serum total cholesterol (mg/dl) | 149.7 ± 34.3 | 159.3 ± 34.7* | 157.7 ± 34.6 | 151.1 ± 35.3 |
| Serum HDL cholesterol (mg/dl) | 53.8 ± 16.7 | 59.3 ± 17.1† | 57.7 ± 16.7 | 56.4 ± 18.3 |
| Plasma glucose (mg/dl) | 106.1 ± 28.5 | 98.3 ± 20.8† | 101.5 ± 25.9 | 99.8 ± 18.1 |
| Serum creatinine (mg/dl) | 1.65 ± 0.51 | 1.30 ± 0.43§ | 1.45 ± 0.48 | 1.33 ± 0.51* |
| eGFR (ml/min/1.73 m2) | 48.4 ± 22.2 | 65.7 ± 25.1§ | 57.0 ± 23.2 | 66.8 ± 29.9† |

Abbreviations: mRAP, mean right atrial pressure; mPAP, mean pulmonary arterial pressure; mPCWP, mean pulmonary capillary wedge pressure; HDL, high-density lipoprotein; eGFR, glomerular filtration rate estimated from serum creatinine. Heart rate refers to the heart rate measured during right heart catheterization. Office heart rate was measured on the day of the urine collection within 6 months of the catheterization. An office heart rate of 88 corresponded to the 75th percentile of the distribution. For years since transplantation the median (interquartile range) is given. Hypertension was an office blood pressure of at least 140 mmHg systolic or 90 mmHg diastolic or use of antihypertensive drugs. Diabetes mellitus was a hospital diagnosis, a fasting plasma glucose of 126 mg/dl or higher, or use of antidiabetic agents. Elevated right heart pressure was mRAP (≥10 mm Hg), mPAP (≥24 mm Hg), or mPCWP (≥17 mm Hg) equal to or exceeding the 75th percentile of the distributions. Significance of the between-group difference: * p ≤ 0.05; † p ≤ 0.01; ‡ p ≤ 0.001; § p ≤ 0.0001.
