# Supplementary material for: Urinary proteomic signatures associated with β-blockade and heart rate in heart transplant recipients
Source: PLoS One. 2018 Sep 24;13(9):e0204439. doi: 10.1371/journal.pone.0204439 (PMC6152976; doi:10.1371/journal.pone.0204439)
Supplement: S6 Table — (DOCX) [file pone.0204439.s006.docx]

**S6 Table.**

**Use of medications by β-blocker use or office heart rate**

| **Characteristic** | **Use** | **Non-use** | **<88 beats/minute** | **≥88 beats/minute** |
| --- | --- | --- | --- | --- |
| Number of participants (%) | 118 | 218 | 245 | 91 |
| Immunosuppressive treatment |  |  |  |  |
| Calcineurin inhibitor | 115 (97.5) | 212 (97.3) | 238 (97.1) | 89 (97.8) |
| Tacrolimus | 80 (67.8) | 180 (82.6)† | 187 (76.3) | 73 (80.2) |
| Cyclosporine | 35 (29.7) | 32 (14.7)‡ | 51 (20.8) | 16 (17.6) |
| Antiproliferative agents | 98 (83.1) | 189 (86.7) | 203 (82.9) | 84 (92.3)* |
| mTOR inhibitors | 8 (6.8) | 11 (5.1) | 13 (5.3) | 6 (6.6) |
| Methylprednisolone | 42 (35.6) | 80 (36.7) | 84 (34.3) | 38 (41.8) |
| Antihypertensive drugs |  |  |  |  |
| Any drug class | 82 (69.5) | 140 (64.2) | 173 (70.6) | 67 (73.6) |
| β-blockers | 118 (100) | 0 (0) … | 95 (38.8) | 23 (25.3)* |
| Thiazides | 21 (17.8) | 14 (6.4)‡ | 27 (11.0) | 8 (8.8) |
| Loop diuretics | 10 (8.5) | 24 (11.0) | 23 (9.4) | 11 (12.1) |
| Aldosterone antagonists | 4 (3.4) | 16 (7.3) | 14 (5.7) | 6 (6.6) |
| Calcium channel blockers | 31 (26.3) | 68 (31.2) | 67 (27.4) | 32 (35.2) |
| RAS inhibitors | 64 (54.2) | 84 (38.5)† | 114 (46.5) | 34 (37.4) |
| Use of statins | 112 (94.9) | 202 (92.7) | 228 (93.1) | 86 (94.5) |
| Use of antidiabetic drugs |  |  |  |  |
| Insulin | 19 (16.1) | 16 (7.3)* | 23 (9.4) | 12 (13.2) |
| Other agents | 22 (18.6) | 31 (14.2) | 34 (13.9) | 19 (20.9) |

Abbreviations: mTOR, mammalian target of rapamycin; RAS renin-angiotensin system. Drugs by class: calcineurin inhibitors, tacrolimus and cyclosporine; antiproliferative agents, azathioprine and mycophenolate mofetil; mTOR inhibitors, everolimus and sirolimus; RAS inhibitors, converting-enzyme inhibitors and angiotensin II type‑1 receptor blockers. An office heart rate of 88 beats/minute corresponded to the 75th percentile of the distribution. Significance of the between-group differences: … p not computed; * p ≤ 0.05; † p ≤ 0.01; ‡ p ≤ 0.001; § p ≤ 0.0001.
