# Supplementary material for: Urinary proteomic signatures associated with β-blockade and heart rate in heart transplant recipients
Source: PLoS One. 2018 Sep 24;13(9):e0204439. doi: 10.1371/journal.pone.0204439 (PMC6152976; doi:10.1371/journal.pone.0204439)
Supplement: S7 Table — (DOCX) [file pone.0204439.s007.docx]

**S7 Table.**

**Urinary levels of classifiers by β‑blocker use or office heart rate**

| Model   Classifier |  | **β‑blocker use *vs.* non-use** | | | |  | **Heart rate categories** | | | |
| --- | --- | --- | --- | --- | --- | --- | --- | --- | --- | --- |
|  |  | Use | Non-use | Δ (95% CI) | *p* |  | <88 bpm | ≥88 bpm | Δ (95% CI) | *p* |
| Adjusted |  |  |  |  |  |  |  |  |  |  |
| HF1 |  | –0.53 ± 0.09 | –0.78 ± 0.06 | –0.25 (–0.46, –0.03) | 0.024 |  | –0.66 ± 0.06 | –0.77 ± 0.10 | 0.11 (–0.11, 0.33) | 0.32 |
| HF2 |  | 0.07 ± 0.06 | –0.12 ± 0.04 | –0.19 (–0.34, –0.05) | 0.009 |  | –0.04 ± 0.04 | ­­–0.10 ± 0.06 | 0.06 (–0.08, 0.21) | 0.40 |
| ACSP75 |  | 0.47 ± 0.24 | –0.11 ± 0.18 | –0.59 (–1.20, 0.02) | 0.060 |  | 0.01 ± 0.16 | 0.31 ± 0.27 | –0.30 (–0.93, 0.33) | 0.35 |
| CKD273 |  | 0.19 ± 0.04 | 0.07 ± 0.03 | –0.12 (–0.21, –0.02) | 0.017 |  | 0.13 ± 0.03 | 0.05 ± 0.04 | 0.09 (–0.01, 0.19) | 0.082 |
| Mutually  adjusted |  |  |  |  |  |  |  |  |  |  |
| HF1 |  | –0.79 ± 0.07 | –0.56 ± 0.09 | –0.24 (–0.45, –0.02) | 0.029 |  | –0.63 ± 0.06 | –0.72 ± 0.10 | 0.09 (–0.13, 0.31) | 0.41 |
| HF2 |  | –0.13 ± 0.04 | 0.06 ± 0.06 | –0.19 (–0.33, –0.04) | 0.011 |  | –0.01 ± 0.04 | –0.06 ± 0.07 | 0.05 (–0.10, 0.20) | 0.52 |
| ACSP75 |  | –0.04 ± 0.19 | 0.57 ± 0.26 | –0.61 (–1.22, –0.01) | 0.049 |  | 0.09 ± 0.17 | 0.44 ± 0.28 | –0.35 (–0.98, 0.28) | 0.28 |
| CKD273 |  | 0.06 ± 0.03 | 0.16 ± 0.04 | –0.11 (–0.20, –0.01) | 0.028 |  | 0.15 ± 0.03 | 0.07 ± 0.04 | 0.07 (–0.02, 0.17) | 0.14 |

Values are mean ± SE or mean between-group differences (Δ) with 95% confidence interval (95% CI). All models were adjusted for time since transplantation, age, mean arterial pressure, body mass index, total-to-HDL cholesterol ratio and the presence of diabetes mellitus. For HF1, HF2 and ACSP75, models were additionally adjusted for glomerular filtration rate estimated from serum creatinine. Mutually adjusted models included both β−blocker use *vs.* non-use and office heart rate categorized by 88 beats per minute (bpm), the 75th percentile of the distribution.
