# Supplementary material for: Urinary proteomic signatures associated with β-blockade and heart rate in heart transplant recipients
Source: PLoS One. 2018 Sep 24;13(9):e0204439. doi: 10.1371/journal.pone.0204439 (PMC6152976; doi:10.1371/journal.pone.0204439)
Supplement: S8 Table — (DOCX) [file pone.0204439.s008.docx]

**S8 Table.**

**Urinary levels of classifiers by use vs. non-use of inhibitors of the renin-angiotensin system**

| Model   Classifier |  | **RAS inhibitor use vs. non-use** | | | |
| --- | --- | --- | --- | --- | --- |
|  |  | Use | Non-use | Δ (95% CI) | *p* |
| Adjusted |  |  |  |  |  |
| HF1 |  | –0.45 ± 0.07 | –0.88 ± 0.07 | –0.44 (–0.64, –0.24) | <0.0001 |
| HF2 |  | 0.24 ± 0.05 | –0.29 ± 0.04 | –0.53 (–0.66, –0.41) | <0.0001 |
| ACSP75 |  | 0.42 ± 0.22 | –0.17 ± 0.19 | –0.60 (–1.18, –0.01) | 0.046 |
| CKD273 |  | 0.27 ± 0.03 | –0.01 ± 0.03 | –0.28 (–0.37, –0.19) | <0.0001 |

Inhibitors of the renin-angiotensin system include angiotensin-converting enzyme inhibitors and blockers of the type‑1 angiotensin II receptor. Values are mean ± SE or mean between-group differences (Δ) with 95% confidence interval (95% CI). All models were adjusted for time since transplantation, age, mean arterial pressure, body mass index, heart rate, total-to-HDL cholesterol ratio and the presence of diabetes mellitus. For HF1, HF2 and ACSP75, models were additionally adjusted for glomerular filtration rate estimated from serum creatinine.
